# Endovascular treatment of unruptured intracranial aneurysms at a single center: Outcomes, selection strategy, and transparent communication for patient decision-making

**DOI:** 10.1177/15910199251394476

**Published:** 2025-11-07

**Authors:** Gianmarco Bernava, Abiram Sandralegar, Jeremy Hofmeister, Andrea Rosi, Hasan Yilmaz, Sandrine Morel, Philippe Reymond, Olivier Brina, Michel Muster, Karl-Olof Lovblad, Karl Schaller, Philippe Bijlenga, Paolo Machi

**Affiliations:** 1 Division of Neuroradiology, 27230 Geneva University Hospitals, Geneva, Switzerland; 2 Division of Neurosurgery, 27230 Geneva University Hospitals, Geneva, Switzerland; 3 HUG NeuroCentre, Geneva University Hospitals, Geneva, Switzerland; 4 Department of Radiology, Mayo Clinic, Rochester, Minnesota, USA

**Keywords:** Endovascular treatment, outcomes, patient informed decision-making, unruptured intracranial aneurysm

## Abstract

**Background:**

The management of unruptured intracranial aneurysms (UIA) requires a balance between procedural risks and the potential benefit of rupture prevention.

**Objective:**

The aim of this study is to evaluate the clinical and anatomical results of endovascular treatment for UIAs at our institution, to benchmark our practice and potential areas for improvement, and enable transparent communication with patients by providing accurate, evidence-based information.

**Methods:**

We reviewed all patients treated for an UIA between January 2017 and July 2022. Patients were grouped according to treatment technique: simple or balloon-assisted coiling; stent-assisted coiling; or flow-diverter stent placement. Clinical outcomes included perioperative mortality, transient (<3 months), and permanent morbidity (>3 months). Anatomical results were graded with the Raymond-Roy or the O’Kelly-Marotta scales. Retreatment rates, length of hospitalization, readmissions, irradiation time, and total radiation dose were assessed.

**Results:**

In total, 169 patients with 201 UIAs underwent 187 endovascular procedures without death, 1% permanent morbidity, 8% transient morbidity, and 4% retreatment. The treatment subgroups analysis shows an absence of permanent morbidity associated with flow-diverter stenting and stent-assisted coiling. Simple/balloon-assisted coiling suffered 2.6% permanent morbidity. Transient morbidity was observed in 9%, 9.5%, and 6.6% of flow-diverter stenting, stent-assisted coiling, and simple/balloon-assisted coiling, respectively.

**Conclusions:**

In our hospital, about one in a hundred procedures caused lasting problems, while about one in 25 aneurysms needed a second treatment. These results reflect our case selection and multidisciplinary approach. Reporting them transparently helps patients understand what outcomes to expect at our hospital.

## Introduction

Unruptured intracranial aneurysms (UIAs) pose a complex therapeutic challenge, balancing procedural risk with rupture prevention.^1,2^ Endovascular treatment has become a cornerstone in constant evolution,^3,4^ yet clinical and anatomical results vary based on case complexity and technique.

The objective of this study was to assess how UIAs were treated endovascularly at our center, to understand our outcomes in the context of the literature, and to provide patients with realistic, local data to support treatment decisions.

## Methods

We conducted a retrospective analysis of our institution's prospectively maintained database to review all consecutive patients who underwent endovascular treatment for UIAs between January 2017 and July 2022. This period marks the establishment of our interventional neuroradiology team. The goal was to assess clinical and anatomical outcomes in our patient cohort. To assess outcomes based on the technical complexity of the endovascular treatment, we categorized the patient cohort into three groups: treated with (1) flow-diverter stent; (2) stent-assisted coiling; and (3) simple- or balloon-assisted coiling. Treatment modality was not randomized but decided in a multidisciplinary board, based mainly on aneurysm location and shape (aspect ratio). Complex MCA aneurysms were usually referred for clipping, while carotid and posterior circulation aneurysms were more often treated endovascularly.^5^

Clinical outcomes included perioperative mortality and morbidity. Morbidity was classified as transient when neurological symptoms, deficits or procedure-related complications resolved within 3 months, and as permanent when they persisted beyond this period. Functional status was systematically assessed using the disability modified Rankin Scale (mRS) ranging from 0 (no symptoms) to 6 (death).

Anatomical outcomes included: aneurysm occlusion rates determined using the Raymond-Roy classification^6^ with grade 1 meaning a completely sealed aneurysm and grade 3 a remaining aneurysm filling after coiling, and the O’Kelly-Marotta scale^7^ for those treated with FD stents, with class D corresponding to complete occlusion of the aneurysm. Additionally, we analyzed hospitalization duration, readmission, and retreatment rates, as well as procedural factors, such as irradiation time and total radiation dose delivered to the patient. This study assessing the quality of procedures and outcomes retrospectively was deemed to fall outside the scope of Swiss human research legislation and consequently did not require approval by a local ethics committee. The study protocol was registered prior before starting the study.

## Statistical analysis

Descriptive statistical analyses were performed to summarize the variables under investigation. Continuous variables were reported as mean and standard deviation (mean ± SD). Discrete variables as median and interquartile range (median,[IQR]). Additionally, percentiles were calculated to illustrate the proportion of specific categories within the total sample. Proportions are reported with 95% confidence intervals calculated using the Wilson score method (%, [95%CI].

## Results

Between January 2017 and July 2022, a total of 465 patients with 497 intracranial aneurysms were treated at our center. Of these, 315 aneurysms underwent endovascular treatment, while 182 underwent surgical intervention. Among the aneurysms treated with endovascular treatment, 201 were unruptured and 114 were ruptured. Almost all unruptured middle cerebral artery aneurysms were treated surgically (90/92, 97.8%, [94.8–100%]), most anterior communicating artery aneurysms were treated endovascularly (41/64, 64.1%, [52.3–75.8%]), almost all posterior circulation and posterior communicating aneurysms were treated endovascularly (51/53, 96.2%, [91.1–100%]). All proximal intracranial carotid aneurysms were treated endovascularily (80/80, 100%, [95.5–100%]). Factors influencing the choice of treatment modality are the aneurysm location mostly and the aspect ratio. Among the 169 patients harboring 201 UIAs, 135 were female (mean patient age, 57 years [±11 years]).

In 187 procedures there were no deaths, 2 lasting complications, 15 temporary complications, 8 retreatments, and an average 3-night hospital stay (Tables 1 and 2). A total of 91 procedures with flow-diverter stents were performed in 81 patients, treating 100 aneurysms. A total of 21 procedures with stent-assisted coiling were performed in 20 patients, treating 21 aneurysms. A total of 75 procedures with simple or balloon-assisted coiling stents were performed in 60 patients, treating 80 aneurysms. Follow-up data were available for 155/169 (92%) patients. Mean follow-up duration was 21 ± 13 months and retreatment was required in 8/201 (4%, [2–8%]) aneurysms. Notably, no patients required readmission due to clinical complications after discharge.

**Table 1.** Location of unruptured intracranial aneurysms and treatments.

Aneurysm location	Endovascular n (%, 95% CI)	Surgery n (%, 95%CI)	SAC&BAC n (95% CI in %)	SAC n (95% CI in %)	FDS n (95% CI in %)
Carotido-ophtalmic	67 (33%, 27.2–40.1%)	0 (0%, 0–2.5%)	4 (2.0–12.2%)	0 (0–15.4%)	63 (53.2–71.9%)
Anterior communicating artery	41 (20.5%, 15.4–26.5%)	23 (19.3%, 12.2–26.4%)	29 (26.7–47.1%)	12 (37.7–74.3%)	0 (0–3.7%)
Posterior communicating artery	21 (10.5%, 6.9–15.4%)	2 (1.7%, 0.2–6.0%)	10 (6.9–21.6%)	0 (0–15.4%)	11 (6.3–18.5%)
Basilar artery tip	16 (8%, 5.0–12.5%)	0 (0%, 0-2.5%)	13 (9.8–25.9%)	2 (2.6–28.9%)	1 (0.2–5.4%)
Carotido-cave	13 (6.5%, 3.8–10.7%)	0 (0%, 0–2.5%)	0 (0–4.6%)	0 (0–15.4%)	13 (7.7–21.1%)
Carotid terminus	10 (5%, 2.8–8.9%)	0 (0%, 0–2.5%)	6 (3.5–15.4%)	2 (2.6–28.9%)	2 (0.6–7.0%)
Anterior choroidal artery	9 (4.5%, 2.4–8.2%)	2 (1.7%, 0.2–6.0%)	3 (1.3–10.6%)	0 (0–15.4%)	6 (2.8–12.5%)
Basilar trunk	7 (3.5%, 1.7–7.0%)	0 (0%, 0–2.5%)	3 (1.3–10.6%)	2 (2.6–28.9%)	2 (0.6–7.0%)
Anterior cerebral artery A1	6 (3%, 1.4–6.4%)	0 (0%, 0–2.5%)	5 (2.7–14.0%)	1 (0.9–22.7%)	0 (0–3.7%)
Middle cerebral artery	2 (1%, 0.3–3.6%)	90 (75.6%, 67.9–83.4)	2 (0.7–8.6%)	0 (0–15.4%)	0 (0–3.7%)
Others	9 (4.5%, 2.4–8.2%)	2 (1.7%, 0.2–6.0%)	5 (2.7–14.0%)	2 (2.6–28.9%)	2 (0.6–7.0%)
Total	201 (100%)	119 (100%)	80 (40%)	21 (10%)	100 (50%)

Abbreviations: SC: simple coiling; BAC: balloon-assisted coiling; SAC: stent-assisted coiling; FDS: flow-diverter stent.

**Table 2.** Endovascular procedures and outcome by technique.

	SC&BAC	SAC	FDS	Total
Number of procedures performed (n)	**75**	**21**	**91**	**187**
Mortality (n) % (95% CI)	0 0% (0.0–4.9%)	0 0% (0.0–15.5%)	0 (0%, 0.0–4.1%)	0 0% (0.0–2.0%)
Permanent morbidity (n) % (95% CI)	2 2.7% (0.7–9.2%)	0 0% (0.0–15.5%)	0 (0%, 0.0–4.1%)	2 1.1% (0.3–3.8%)
Transient morbidity (n) % (95% CI)	5 6.7% (2.9–14.7%)	2 9.5% (2.7–28.9%)	8 (9%, 4.5–16.4)	15 8.0% (4.9–12.8%)
Neurological morbidity (n) % (95% CI)	4 5.3% (2.1–12.9%)	2 9.5% (2.7–28.9%)	4 (4.4%, 1.7–10.8%)	10 5.3% (2.9–9.6%)
Ischemic complications (n) % (95% CI)	3 4% (1.4–11.1%)	2 9.5% (2.7–28.9%)	2 (2.2%, 0.6–7.7%)	7 3.7% (1.8–7.5%)
Hemorragic complications (n) % (95% CI)	1 1.3% (0.2–7.2%)	0 0% (0.0–15.5%)	0 (0%, 0.0–4.1%)	1 0.5% (0.1–3.0%)
Ponction site complications(n) % (95% CI)	3 4% (1.4–11.1%)	0 0% (0.0–15.5%)	1 (1.2%, 0.2–6.0%)	4 2.1% (0.8–5.4%)
Other complications (n) % (95% CI)	0 0% (0.0–4.9%)	0 0% (0.0–15.5%)	3 (3.3%, 1.1–9.2%)	3 1.6% (0.5–4.6%)
X-ray: Gy cm2 (mean (SD))	122 (50)	153.5 (49)	133 (66)	127 (56)
Irradiation time min. (mean (SD))	41(17)	48 (17)	28 (15.5)	35 (17)
Hospitalization nights (mean (SD))	3 (1.7)	4 (2.6)	3.8 (2.8)	3 (2)
	**SC&BAC**	**SAC**	**FDS**	**Total**
Number of aneurysms (n)	**80**	**21**	**100**	**201**
Aneurysm height mm (mean (SD))	6 (3.9)	4.5 (1.6)	6.5(4)	6.1 (3.8)
Aneurysm diameter mm (mean (SD))	5.8 (4.5)	4.7 (1.6)	6.5 (4)	6 (4)
Neck mm (mean (SD))	3.5 (1.6)	3.8 (1.5)	4 (2.5)	3.8 (2.4)
Dome to neck ratio (mean (SD))	1.7 (0.7)	1 (0.5)	1.7 (0.7)	1.6 (0.7)
Occlusion after EVT median	2 (1–3a)^a^	2 (1–3a)^a^	A = 95%^b^ B = 5%^b^	n.a.
Occlusion follow-up median	1 (1- 2)^a^	1 (1- 1)^a^	A = 4%^b^ B = 3%^b^ C = 20%^b^ D = 72%^b^ n.a. = 1%	n.a.
Retreatment (n) % (95% CI)	4 5% (2.0–12.2%)	0 0% (0.0–15.5%)	4 4% (1.6–9.8%)	8 4% (2.0–7.7%)
Follow-up months (mean (SD))	18 (9)	18 (12)	23 (16)	21 (13)

Abbreviations: SC: simple coiling; BAC: balloon-assisted coiling; SAC: stent-assisted coiling; FDS: flow-diverter stent; SD: standard deviation.

^a^According to the Raymond-Roy scale (IQR). IQR: interquartile range.

^b^According to O'Kelly-Marotta scale.

The patient death after endovascular treatment was observed (0%, [0–2%]). The intervention rate of permanent morbidity was 2/187 (1%, [0.3–3.8%]). The rate of transient morbidity 15/187 (8%, [4.9–12.8%]). The neurological morbidity rate associated with interventions was 10/187 (5.3%, [2.6–9.6%]).

The average length of hospitalization was 3 ± 2 nights and the mean irradiation time was 35 ± 17 min. It was comparable for balloon-assisted coiling/simple coiling and stent-assisted coiling (41 ± 17 min and 48 ± 17 min, respectively), but considerably lower for patients treated with flow-diverter stents (28 min ± 15.5). The average radiation dose delivered to patients was 127 ± 56 Gy·cm² (Table 2).

## Outcomes by procedural technique

**Figure 1.**
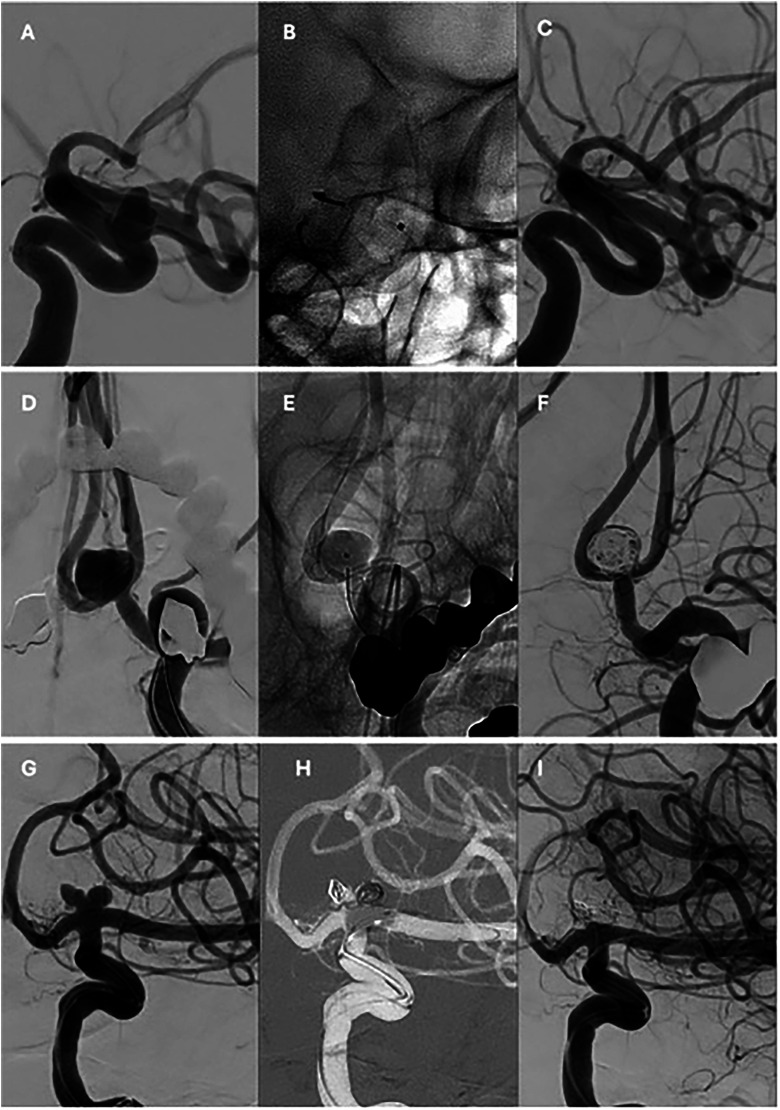
(A–C) Lateral angiographic projections of the endovascular treatment of a left ophthalmic carotid aneurysm with a flow-diverter stent. (A) Pre-treatment angiogram. (B) Unsubtracted image showing the implanted device. (C) Six-month follow-up angiogram. (D–F) Anteroposterior angiographic projections of a recurrent anterior communicating artery aneurysm previously treated with surgical clipping 10 years earlier. (D) Pre-treatment angiogram. (E) Unsubtracted image showing the braided stent and a jailed microcatheter within the aneurysm sac. (F) Final post-treatment angiogram. (G–I) Anteroposterior angiographic projections of the endovascular treatment of a left internal carotid artery terminus aneurysm. (G) Pre-treatment angiogram. (H) Coil deployment within the aneurysm sac assisted by a remodeling balloon. (I) Final angiographic result.

*Aneurysms treated with flow-diverter stents.* A total of 91 interventions for the treatment of 100 UIAs were performed (Figure 1(a)-(c)). Among these procedures, no mortality or permanent morbidity was observed (Table 2). Transient morbidity occurred in 8/91 interventions (9%,[4.5–16.4%]) with one-half of patients (n = 4) presenting transient neurological deficits upon awakening only. Among them, one developed contrast-induced encephalopathy, which resolved within 24 h. Two cases of embolic stroke were recorded, with complete neurological recovery within 3 months. Additionally, one patient experienced acute thrombosis of an intracavernous aneurysm, manifesting as facial pain and diplopia, with full resolution of symptoms before the 3-month follow-up.

Transient morbidity in four other patients was related to non-neurological complications. One patient developed hematomas at the puncture site, successfully managed with simple compression. Two patients with iatrogenic asymptomatic internal cervical carotid artery dissection were treated with a stent, while one patient experienced transient hematuria.

The mean follow-up period was 23 ± 16 months. At the last follow-up, 72% of patients had a complete occlusion of the aneurysm while 20% had an entry remnant, according to the O’Kelly-Marotta scale. Retreatment was necessary in four cases (4%,[1.6–9.8%]) due to a major recurrence. These included two posterior communicating artery aneurysms, one giant carotid-ophthalmic aneurysm, and one dissecting aneurysm of the basilar artery.

*Aneurysms treated with stent-assisted coiling*. A total of 21 interventions for the treatment of 21 UIAs were performed with stent-assisted coiling (Table 2) (Figure 1(d)–(f)). No mortality or permanent morbidity was observed (0%, [0–15.5%]). Transient morbidity occurred in 2/21 interventions (9.5%, [2.7–28.9%]) due to embolic strokes. However, all neurological symptoms resolved within 3 months. The mean follow-up duration was 18 ± 12 months. Median aneurysm occlusion according to the Raymond-Roy scale was 2[1–3a] at the end of the procedure, improving to 1[1–1] at the last follow-up. No cases required retreatment.

*Aneurysms treated with simple- or balloon-assisted coiling.* A total of 75 interventions for the treatment of 80 UIAs were performed using simple- or balloon-assisted coiling (Table 2) (Figure 1(g)–(i)).

No mortality was recorded (0%,[0–4.9%]), while permanent morbidity occurred in 2/75 interventions (2.7%, [0.7–9.2%]). These cases were attributed to one hemorrhagic and one ischemic complication. The hemorrhagic complication resulted from microguidewire perforation of the posterior inferior cerebellar artery during the treatment of the aneurysm at its origin. Arterial occlusion was necessary, leading to cerebellar ischemia. The patient subsequently experienced persistent hearing and balance deficits (mRS 2). The ischemic complication was caused by a distal embolic stroke in the territory of the left angular artery, resulting in mild aphasia (mRS 2).

Transient morbidity was observed in 5/75 interventions (6.7%,[2.9–14.7%]) with complications arising from embolic stroke (n = 2), retroperitoneal hematoma (n = 2), one of which required surgical intervention, and femoral artery occlusion (n = 1) requiring surgical recanalization. All patients with transient morbidity were asymptomatic at the 3-month clinical follow-up.

The mean follow-up duration was 18 ± 9 months. Median aneurysm occlusion was 2[1–3a] on the Raymond-Roy scale at the end of the procedure, improving to 1[1–2] at the last follow-up. Retreatment was required in 4/75 cases (5.3%,[2–12.2%]) due to significant recurrence. These included one giant aneurysm, one anterior communicating artery aneurysm, one basilar tip aneurysm, and one wide-neck A1 aneurysm in a patient on lifelong oral anticoagulation therapy.

## Discussion

Careful selection between surgical and endovascular modalities, and among endovascular techniques, enabled relatively safe treatment of UIAs in our cohort. We observed no deaths and only 1% permanent neurological morbidity (95% CI 0.3–3.8%) and 4% retreatment rate (95% CI 2–8%). Importantly, other large series have reported mortality or permanent deficit rates up to 3%. Our results are consistent with published series^8,9^ and suggest that, in carefully selected patients, endovascular treatment can be performed with low rates of major complications. It is important to note that these favorable results reflect strong case selection, particularly the systematic referral of MCA aneurysms to surgery, and may not generalize to all centers or aneurysm types.

Results align with the findings of Algra et al.^9^ who performed a meta-analysis of 114 studies involving over 100,000 patients and reported a 30-day complication rate of 4.96%, including a mortality rate of 0.3%, ischemic events in 2.8%, and hemorrhagic events in 0.9% . Notably, despite the anticipated higher risk associated with more complex endovascular techniques, such as stent-assisted coiling and flow-diverter stents, our data revealed no significant increase in morbidity or mortality compared to simple- or balloon-assisted coiling.

With regard to flow-diverter stents, our series reported no mortality or permanent morbidity. This mirrors the low but non-zero risks reported in large meta-analyses such as Bhatia et al.,^8^ which assessed 901 PED Flex procedures and found a mortality rate of 0.8%, a 0.6% rate of major ischemic stroke, and a 1.2% rate of intracranial hemorrhage, culminating in an overall complication rate of 9.8%. Similarly, Zhou et al.^10^ reported a mortality rate of 2.8% and a permanent morbidity rate of 3.7% across more than 3000 patients treated with flow-diverter stents. In our cohort, only two minor strokes (2.2%) and six other complications (6.6%) were observed across 91 procedures. Of particular note, none of our flow-diverter-treated patients experienced major complications.

The use of stent-assisted coiling has traditionally raised concerns due to the need for dual antiplatelet therapy and the theoretical risk of thromboembolic and hemorragic events.^11,12^ Nevertheless, our data showed no mortality or permanent morbidity in this subgroup. Two patients (9.5%) experienced transient ischemic complications with complete recovery within 3 months. These results echo those of Ji et al.^13^ who found that stent-assisted coiling may be protective against neurological complications in selected patients.

However, the literature on this topic remains heterogeneous. Meta-analyses by Cagnazzo^14^ and Akram^15^ reported complication rates of 5–7.4%, with ischemic events in 2.4–2.6%, in-stent thrombosis in 1.5–3.5%, and overall morbidity around 1.5%. A key limitation of these studies is the inclusion of ruptured aneurysms, which are known to carry substantially higher peri-procedural risks. Indeed, Bechan et al.^16^ demonstrated that the presence of subarachnoid hemorrhage increases the risk of thromboembolic events, thereby confounding comparisons with series limited to unruptured aneurysms.

Our findings regarding simple- and balloon-assisted coiling are also consistent with the ATENA study,^17^ which evaluated 739 unruptured aneurysms across 27 centers and reported an overall complication rate of 15.4%, including embolic stroke in 7.1% and procedural rupture in 2.6%, with a mortality rate of 0.9%. In our series, no mortality was recorded in this subgroup and permanent morbidity was limited to 2.5%. In our study, embolic stroke occurred in 4% of cases for this intervention group, but only one patient experienced a lasting neurological deficit. Notably, no cases of aneurysm rupture occurred during the procedures; the single hemorrhagic complication resulted from perforation of an artery during microcatheter navigation.

Importantly, when comparing across treatment modalities, we observed no notable difference in permanent morbidity or retreatment rates.

These favorable outcomes may be partly attributed to the multidisciplinary approach adopted at our center for patient selection. However, such an algorithm is specific to our center, tailored to individual patient characteristics, and therefore not directly generalizable to other institutions or settings (Table 3). It also represents a simplified depiction of our decision-making process, from which we may deliberately deviate in specific cases when clinically justified.

**Table 3.** Decision-making algorithm for unruptured intracranial aneurysms.

**Identify aneurysm location** **Anterior circulation** **Middle cerebral artery bifurcation** → **Microsurgical clipping**! **Pericallosal bifurcation** → **Microsurgical clipping vs Endovascular treatment** **Proximal ICA side-wall** → **Endovascular treatment** **Carotid terminus**→ **Endovascular treatment** **Anterior communicating artery** → **Primarily coiling,** *clipping or stenting when branch preservation is challenging* **Posterior communicating artery or anterior choroidal artery small aneurysms** → **Primarily coiling or stenting**, *clipping when branch preservation is challenging* **Posterior circulation** **Basilar artery** → **Endovascular treatment** **Posterior inferior cerebellar artery** →**Primarily coiling**, *clipping when branch preservation is challenging* **Assess aneurysm morphology** **Narrow neck** → **Simple or balloon-assisted coiling** **Wide neck or low dome-to-neck ratio** → **Flow diversion or clipping or stent-assisted coiling**, depending on location **Evaluate risk–benefit balance** **If procedural risk** **>** **expected benefit** → **Surveillance**

The distribution of aneurysm locations in our cohort is broadly consistent with that reported in the international literature and with data previously published by our group. In the large pooled analysis conducted by the International Stroke Genetics Consortium, the most frequent sites were the anterior communicating artery (26.7%), the middle cerebral artery (25.9%), and the posterior communicating artery (14.7%).^18,19^ In contrast, our endovascular series showed a predominance of carotido-ophthalmic aneurysms (33%), followed by the anterior communicating artery (20.5%) and the posterior communicating artery (10.5%). This pattern likely reflects a therapeutic strategy guided by a multidisciplinary approach in which both aneurysm location and its morphological characteristics play a crucial role in selecting the treatment modality.

In particular, complex middle cerebral artery aneurysms, commonly associated with higher endovascular-related risk or recurrence, were typically referred for surgical clipping following multidisciplinary team discussion. This collaborative process between interventional neuroradiologists and neurosurgeons ensures tailored therapeutic strategies based on aneurysm morphology, location, and patient-specific factors. Our protocol helps early-career operators by making expert reasoning explicit and reproducible: (i) mandatory multidisciplinary team review with a documented indication and alternatives (including surveillance), (ii) locally audited absolute risks with 95% CIs to calibrate consent and expectations, (iii) non-prescriptive selection tendencies that map common aneurysm archetypes to typical techniques, (iv) a standardized peri-procedural pathway (planning checks, intra-op safety steps, radiation monitoring, follow-up), and (v) clear supervision/escalation criteria with routine debriefs. Together, these elements reduce cognitive load, enhance safety, and accelerate deliberate practice toward independent decision-making.

Our study has several limitations. First, the relatively small number of patients. Second, its single-center design, which limits its generalizability and, third, the non-randomized allocation of treatment.

### Teaching points

In a multidisciplinary, selection-driven practice, endovascular treatment of carefully chosen UIAs can be performed with very low major complications (no deaths, 1% permanent morbidity) and modest retreatment needs (4% over 5 years). Technique selection should be individualized, primarily by aneurysm location and morphology and patient factors, rather than dictated by a rigid algorithm; in our cohort, flow diversion and stent-assisted coiling were not associated with higher permanent morbidity than simple/balloon-assisted coiling. For informed consent, patients should receive absolute, locally audited risks (with 95% CIs) alongside literature benchmarks, including the uncertainty around retreatment and morbidity. When estimated procedural risk exceeds expected benefit, surveillance remains appropriate. Ongoing work will formalize this approach into probabilistic decision support using real-world data, with external, prospective validation (ideally in pragmatic comparative studies).

## Conclusions

This study shows the clinical and anatomical results associated with tailored endovascular treatment of carefully selected UIAs at our center. At our center, endovascular treatment of UIAs resulted in no deaths, about 1% permanent morbidity, and 4% retreatment over a mean follow-up of nearly 2 years. These outcomes reflect our multidisciplinary case selection and local practice. By reporting them in clear terms, we aim to support informed patient decision-making and maintain transparent benchmarking of our performance. Continued practice and outcome monitoring will be essential to maintain optimal practices and further improve patient outcomes in this evolving field.
